# The role of psychological factors and food in improving athletic performance: an analytical study of a specific sports organization’s society

**DOI:** 10.3389/fnut.2026.1901744

**Published:** 2026-07-27

**Authors:** Ping Wang, Xu Li

**Affiliations:** 1 College of Competitive Sports, Shandong Sport University, Rizhao, Shandong, China; 2 Sports Department, Rizhao Polytechnic University, Rizhao, Shandong, China

**Keywords:** athletes, competitive anxiety, mental resilience, motivation, nutritional practices, self-perceived performance

## Abstract

**Background:**

Athletic performance reflects the interplay of physical, psychological, and nutritional factors. Although the individual roles of mental and dietary factors are increasingly recognized, their combined relationship with performance within a defined sports organization has rarely been examined.

**Objective:**

This cross-sectional study examined the associations of psychological factors (motivation, anxiety, mental resilience, self-confidence) and self-reported nutritional practices with self-perceived athletic performance among athletes affiliated with a sports organization in Rizhao, Shandong, China (*n* = 100).

**Methods:**

A descriptive, cross-sectional, analytical design was used. One hundred actively competing athletes completed self-report instruments comprising an adapted Athletic Mental Energy Scale and selected Competitive State Anxiety Inventory-2 (CSAI-2R) subscales (motivation, cognitive and somatic anxiety, mental resilience, self-confidence; 5-point Likert scaling), a nutritional practices questionnaire (pre-competition nutrition, hydration, meal regularity, supplement use), and a self-perceived performance rating (0–10). Data were analyzed in SPSS (v26) using descriptive statistics, Pearson correlation, multiple linear regression controlling for age, sport type, and competitive level, and a moderation analysis; statistical significance was set at *p* < 0.05.

**Results:**

Intrinsic motivation showed the strongest positive association with self-perceived performance (*r* = 0.68, *p* < 0.001), followed by self-reported pre-competition nutrition (*r* = 0.62) and mental resilience (*r* = 0.59), whereas cognitive anxiety was negatively associated (*r* = −0.51). In multiple regression, the model explained 61% of the variance (*R*^2^ = 0.61; *F*(6,93) = 24.3, *p* < 0.001), with intrinsic motivation (*β* = 0.42), mental resilience (*β* = 0.31), and pre-competition nutrition (*β* = 0.28) as the strongest independent predictors and cognitive anxiety as a significant negative predictor (*β* = −0.19); supplement use was not significant (*β* = 0.09). Nutritional adherence significantly moderated the stress–performance association (interaction *β* = −0.23, *p* = 0.014), such that higher adherence was associated with a smaller decline in performance at higher stress levels.

**Conclusion:**

Within this organization, psychological factors and nutritional practices were jointly associated with self-perceived athletic performance. Integrated support combining motivational and mental-skills strategies with structured nutritional guidance appears important for athlete development, although the cross-sectional, self-report design precludes causal inference.

## 1 Introduction

Athletic performance has traditionally been understood in physiological terms strength, endurance, speed, and technical skill (1). Yet contemporary sports science treats elite performance as multidimensional, shaped not only by physical capacity but also by psychological and nutritional factors affecting athletes’ readiness, consistency, and long-term development (2), particularly within organized sports settings that provide structured support (3).

The most studied psychological factors are motivation, competitive anxiety, mental resilience, and self-confidence. Intrinsically motivated athletes train more consistently and sustain effort under pressure (4), whereas weak or external motivation predicts burnout and inconsistency. Pre-competition anxiety can disrupt concentration, decision-making, and motor execution (5), although moderate arousal aids performance while excessive arousal impairs it the inverted-U relationship (6). Regulating these states is central to applied sport psychology (7).

Mental resilience is a further correlate of performance, especially at higher competitive levels. Nutrition has a comparably prominent role, with dietary intake closely tied to physical output (8). Beyond energy provision, the composition and timing of intake shape endurance, cognition, recovery, and training adaptation (9). Yet many athletes show limited nutritional knowledge, and poor dietary habits are linked to reduced performance and greater injury risk (10).

The two domains are interrelated: psychological states shape dietary behavior, as stressed athletes may eat irregularly or choose poorly (11), while inadequate nutrition impairs cognition and amplifies negative psychological states. The institutional context further shapes the resources available to athletes (12).

Although both have been studied in athletes, they have largely been investigated in parallel rather than together (13). Sport-psychology links motivation and resilience to competitive outcomes, and sports-nutrition links dietary adequacy and timing to performance (14), with only a smaller literature connecting the two (15). Few studies have examined them jointly within a single, defined organization, where shared training norms, competitive pressures, and support systems may shape both. The present study addresses this gap in athletes affiliated with a sports organization in Rizhao, Shandong, China (16).

This study examined how psychological factors and nutritional practices relate to self-perceived athletic performance in this organization, and which factors are most strongly associated with it. Four research questions were addressed: (1) how psychological factors (motivation, anxiety, mental resilience, self-confidence) relate to performance; (2) how self-reported nutritional practices relate to it; (3) which factors are the strongest independent correlates of performance; and (4) whether nutritional adherence moderates the association between competitive stress and performance.

The following directional hypotheses were proposed.

*H1*: Higher intrinsic motivation, mental resilience, and self-confidence are positively associated with self-perceived performance, whereas higher competitive anxiety is negatively associated.

*H2*: More favorable self-reported nutritional practices are positively associated with self-perceived performance.

*H3*: Nutritional adherence moderates the stress–performance association, such that the negative association between stress and performance is weaker among athletes reporting higher adherence.

## 2 Methods

### 2.1 Study design

A descriptive, cross-sectional, analytical design was used to examine the relationships among psychological factors, nutritional practices, and self-perceived athletic performance within a single sports organization. Data were collected at one time point during an active competitive season, providing a snapshot of these variables rather than tracking change over time. Because the design is cross-sectional and relies on self-reported measures, the analyses characterize associations among variables and do not support causal or intervention-based conclusions.

### 2.2 Participants and sampling

The study was conducted within a sports organization located in Rizhao, Shandong, China, in which athletes train in a structured environment supported by institutional coaching and support systems. Participants represented several sports branches, with the distribution across branches reported in Table 1. The organization was a university-affiliated competitive sports organization with approximately 180 registered athletes across basketball, volleyball, and track and field. The athletes competed mainly at regional and national levels. This setting was selected purposively because it provided institutional access, a range of sports, and a competitive environment relevant to the research questions.

**Table 1:** Internal consistency of study subscales (*n* = 100).

Subscale	No. of items	Cronbach’s *α*	McDonald’s *ω*
Motivation (intrinsic/extrinsic)	10	0.87	0.88
Cognitive anxiety	5	0.82	0.83
Somatic anxiety	5	0.80	0.81
Mental resilience	12	0.89	0.90
Self-confidence	6	0.84	0.85
Nutritional practices (per domain)	4 per domain (16 total)	0.78–0.84	0.79–0.85
Self-perceived performance	1	Not applicable	Not applicable

Participants were registered athletes of the organization. A combined purposive and stratified sampling strategy was used: Purposive selection ensured that participants were actively competing athletes with at least 1 year of competitive experience within the organization, and stratification by sport branch, age group, and competitive level improved representativeness across these subgroups.

Inclusion criteria were: (i) current registration as an athlete with the study organization; (ii) at least 1 year of competitive experience within the organization; (iii) active participation in training and competition during the study period; and (iv) provision of written informed consent. Exclusion criteria were: (i) current injury or medical restriction preventing regular training or competition; (ii) off-season status or no scheduled competition during the data-collection period; and (iii) questionnaires with more than 10% missing responses or clearly invalid response patterns.

The required sample size was estimated *a priori* using software, e.g., G*Power v3.1.9.7 for a multiple linear regression (fixed model, *R*^2^ deviation from zero), assuming a medium effect size (*f*^2^ = 0.15), *α* = 0.05, and statistical power of 0.80, which indicated a minimum of 80 participants. To allow for incomplete or invalid responses, 100 athletes were recruited.

### 2.3 Instruments

Data were collected using three self-report instruments, each addressing a component of the research framework. The psychological and anxiety measures were adapted from previously validated scales, whereas the nutritional and performance measures were developed for this study.

#### 2.3.1 Psychological factors scale

Psychological variables were assessed using an adapted version of the Athletic Mental Energy Scale (AMES) (17) combined with selected subscales of the Competitive State Anxiety Inventory-2 Revised (CSAI-2R) (18). The composite instrument measured four constructs: motivation (10-item subscale of intrinsic and extrinsic motivation); pre-competition anxiety (cognitive and somatic subscales, with higher scores indicating greater anxiety); mental resilience (12-item subscale assessing concentration, recovery from setbacks, and performance under pressure); and self-confidence (6-item subscale). Representative items included: “I feel motivated to improve my performance,” “I worry about making mistakes before competition,” “I can regain focus after a setback,” and “I am confident that I can perform well in competition.

Each subscale was scored on a 5-point Likert metric (1 = Strongly Disagree to 5 = Strongly Agree), and subscale scores were computed as the mean of their constituent items, yielding a possible range of 1–5. Higher scores indicate higher levels of the corresponding construct (motivation, mental resilience, or self-confidence); for the anxiety subscales, higher scores indicate greater cognitive or somatic anxiety. The reliability coefficients reported in this study were calculated in the present sample (Table 2); for reference, prior validation studies of these instruments have reported Cronbach’s alpha values above 0.75 (19).

**Table 2:** Pearson correlation matrix (*n* = 100).

Variable	(1)	(2)	(3)	(4)	(5)	(6)
(1) Motivation	1.00					
(2) Resilience	0.61**	1.00				
(3) Anxiety	−0.43**	−0.38**	1.00			
(4) Nutrition	0.52**	0.47**	−0.29**	1.00		
(5) Hydration	0.44**	0.40**	−0.22*	0.63**	1.00	
(6) Performance	0.68**	0.59**	−0.51**	0.62**	0.48**	1.00

**p < 0.01; *p < 0.05 (two-tailed). The diagonal (1.00) represents self-correlation.

Because the instruments were administered in Chinese, the items were adapted through forward translation by two bilingual researchers, independent back-translation by a third bilingual reviewer, reconciliation of discrepancies by the research team, and preliminary testing with 15 athletes to confirm cultural and contextual equivalence.

#### 2.3.2 Nutritional practices questionnaire

Nutritional behavior was assessed using a study-developed nutritional practices questionnaire designed for this research. The questionnaire comprised 16 items organized into four domains reported in the analyses: pre-competition nutrition, hydration practices, meal regularity, and supplement use. Each item was rated on a 5-point Likert scale, yielding a continuous mean score per domain (range 1–5). A representative item is “I follow a planned meal or snack strategy before competitions or important training sessions.” This questionnaire assessed self-reported nutritional behaviors and perceptions; it did not quantify dietary or energy intake, macronutrient or micronutrient composition, precise meal timing, supplement type or dosage, objective hydration status, or sports-nutrition knowledge.

#### 2.3.3 Self-perceived athletic performance measure

Athletic performance was assessed by athlete self-report. Participants rated their own competitive performance on a single-item scale ranging from 0 to 10, with higher scores indicating better self-perceived performance. The item was developed for this study and asked: “Overall, how would you rate your recent competitive performance?.” Because this outcome reflects athletes’ subjective appraisal rather than objective competition results or coach evaluation, it is referred to throughout as self-perceived performance.

#### 2.3.4 Scale access and replication

To improve transparency and support future replication, information on the standardized psychological scales used in this study is provided through their original published sources. The Athletic Mental Energy Scale (AMES) can be accessed through the original AMES development and validation article by Lu et al. (17), and the Chinese validation of AMES can be accessed through the published Chinese validation study. The Competitive State Anxiety Inventory-2 Revised (CSAI-2R) can be accessed through its original validation source and the ePROVIDE/MAPI Research Trust instrument record.

The nutritional practices questionnaire and the single-item self-perceived athletic performance rating were developed for the present study and are not externally available standardized scales. To support interpretation and partial replication, their domains, item numbers, response format, scoring approach, and representative items are described in Sections 2.3.2 and 2.3.3.

### 2.4 Validity and reliability

**Figure 1:**
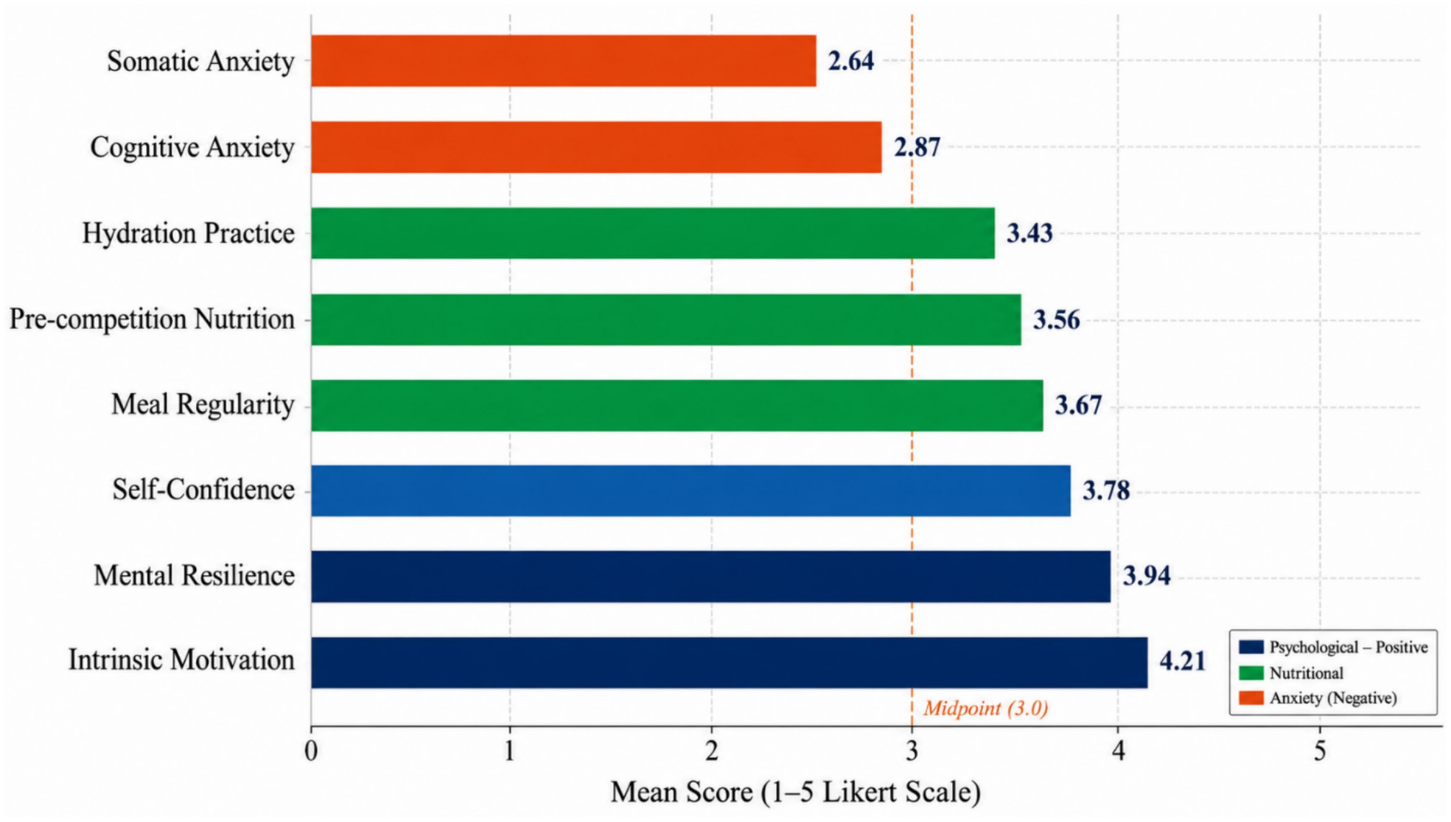
Mean scores across psychological and nutritional variables.

Content validity was established by a panel of three expert reviewers (a sport psychologist, a sports nutritionist, and a senior sports coach), who evaluated all items for relevance, clarity, and consistency with the study constructs; Construct validity was supported by selecting instruments with established theoretical foundations and prior validation. A preliminary study with 15 athletes outside the main sample confirmed item clarity and usability, and minor revisions were made before final administration (Figure 1).

Internal consistency for each subscale was evaluated in the present sample using Cronbach’s alpha and McDonald’s omega, with values ≥ 0.70 considered acceptable, ≥ 0.80 good, and ≥ 0.90 excellent. These indices assess the degree to which items within a subscale measure a common construct and are reported per subscale in the internal consistency table. Internal consistency was estimated separately from any assessment of temporal stability. Because data were collected at a single time point, test–retest reliability was not assessed, and no intraclass correlation coefficients were reported.

### 2.5 Data collection procedure

Following organizational approval, data were collected over 3 weeks during the active competitive season, when participants were competing rather than in the off-season. Questionnaires were administered in paper form during group sessions (team meetings or post-training), with researchers present to clarify items and standardize conditions. Participation was voluntary and confidential, with the right to withdraw at any time without penalty, and all participants provided written informed consent before data collection.

### 2.6 Data analysis

Data were entered and analyzed in IBM SPSS Statistics (Version 26). Prior to inferential analysis, the distribution of continuous variables was examined for normality using the Shapiro–Wilk test and inspection of skewness and kurtosis, and values fell within acceptable limits, supporting the use of parametric tests. Descriptive statistics (means, standard deviations, frequencies, percentages) characterized the sample and the distribution of scores. Pearson correlation examined the direction and strength of bivariate relationships. Multiple linear regression identified the strongest independent predictors of self-perceived performance, controlling for age, sport type, and competitive level. Independent-samples *t*-tests and one-way ANOVA tested for differences across sport type, competitive level, and gender. Statistical significance was set at *p* < 0.05 for all analyses.

### 2.7 Ethical considerations

The study involved human participants and was conducted in accordance with the Declaration of Helsinki and the ethical principles governing research with human participants, including informed consent, voluntary participation, confidentiality, and the right to withdraw. Ethical approval was granted by the Research Ethics Committee of Shandong Sport University (approval/protocol number SSU-REC-2026-001). No personally identifiable information was recorded, and data were stored securely with access restricted to the research team. The study posed no physical, psychological, or social risk to participants, and all participants provided written informed consent before data collection.

## 3 Results

### 3.1 Sample characteristics

The demographic and training characteristics of the 100 participants are summarized in Table 3.

**Table 3:** Demographic and training characteristics of participants (*n* = 100).

Characteristic	Category	*n* (%) or *M* ± SD
Age (years)	—	20.8 ± 2.1 (range 18–26)
Sex	Male	58 (58%)
	Female	42 (42%)
Sport branch	Basketball	35 (35%)
	Volleyball	30 (30%)
	Track and field	35 (35%)
Competitive level	Regional	64 (64%)
	National	36 (36%)
Training experience (years)	—	5.1 ± 2.4
Weekly training frequency (sessions/week)	—	5.7 ± 1.3

### 3.2 Descriptive statistics

Descriptive statistics for all study variables are presented in Table 4. Mean scores for the psychological constructs were generally high, with intrinsic motivation the highest (*M* = 4.21, SD = 0.58) and cognitive and somatic anxiety at moderate levels (*M* = 2.87 and 2.64, respectively) (20). Among the nutritional variables, self-reported pre-competition nutrition was highest (*M* = 3.56), and the mean self-perceived performance score was 7.12 of 10 (SD = 1.43) (21), indicating generally favorable self-appraised performance with some variability across the sample (22).

**Table 4:** Descriptive statistics for all study variables (*n* = 100).

Variable	*n*	Mean	SD	Min	Max
Intrinsic motivation	100	4.21	0.58	2.40	5.00
Cognitive anxiety	100	2.87	0.71	1.00	4.80
Somatic anxiety	100	2.64	0.68	1.00	4.60
Mental resilience	100	3.94	0.62	2.20	5.00
Self-confidence	100	3.78	0.74	1.80	5.00
Pre-competition nutrition	100	3.56	0.81	1.20	5.00
Hydration practice	100	3.43	0.77	1.00	5.00
Meal regularity	100	3.67	0.69	1.60	5.00
Overall self-perceived performance score	100	7.12	1.43	3.50	10.00

Scores for psychological and nutritional variables range from 1 to 5; the performance score ranges from 0 to 10. SD, standard deviation.

### 3.3 Correlation analysis

Bivariate relationships among the key variables were examined using Pearson correlation (Table 2) (23). The variables most strongly and positively correlated with self-perceived performance were intrinsic motivation (*r* = 0.68, *p* < 0.001), pre-competition nutrition (*r* = 0.62), and mental resilience (*r* = 0.59). Cognitive anxiety was significantly negatively correlated with performance (*r* = −0.51) (24).

**Figure 2:**
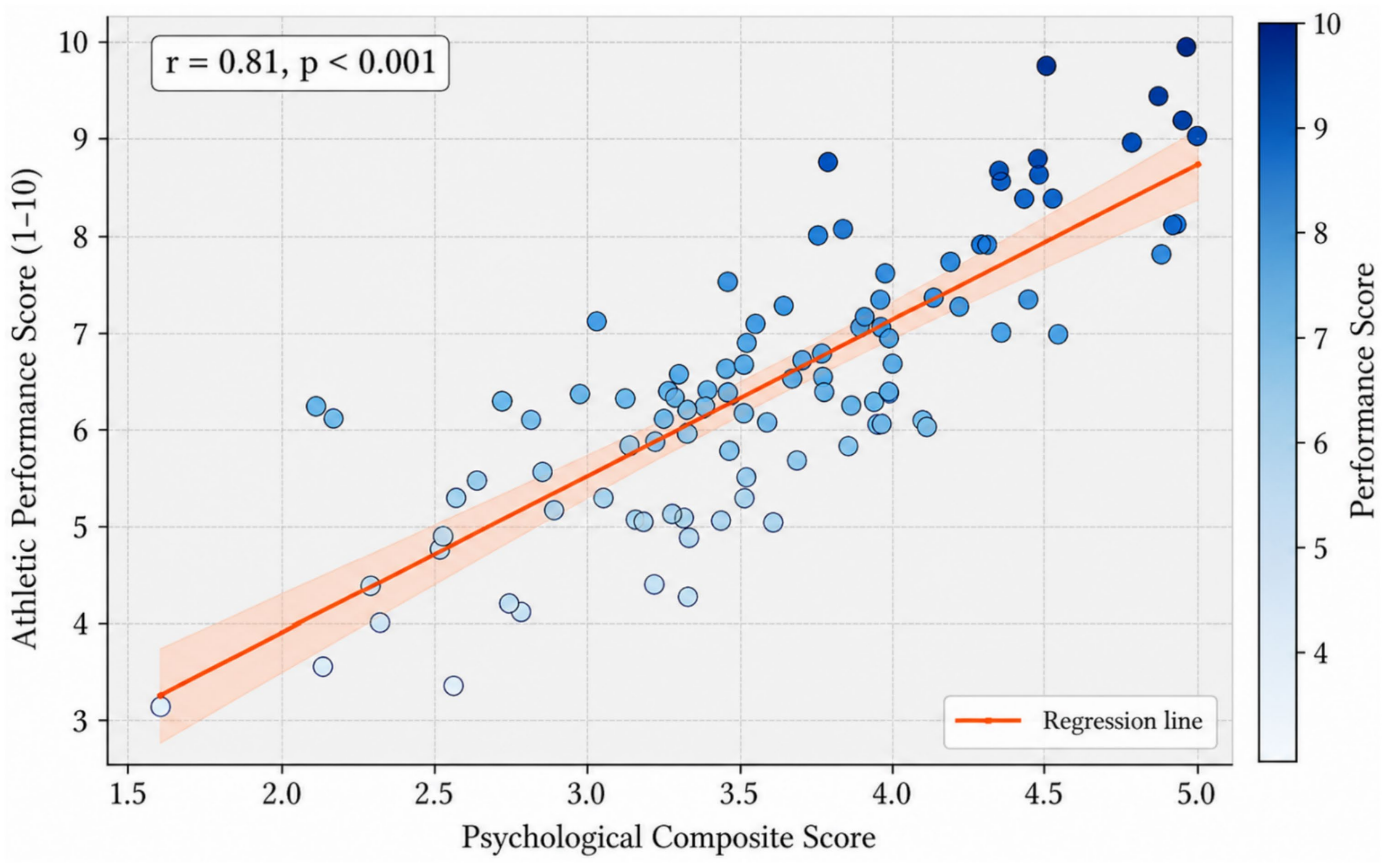
Scatter plot: composite psychological score versus self-perceived athletic performance (*r* = 0.74, *p* < 0.001).

A composite psychological score was also strongly and positively correlated with self-perceived performance (*r* = 0.74, *p* < 0.001), as illustrated by the scatter plot in Figure 2; the regression line and confidence band show a consistent positive slope across the range of scores.

**Figure 3:**
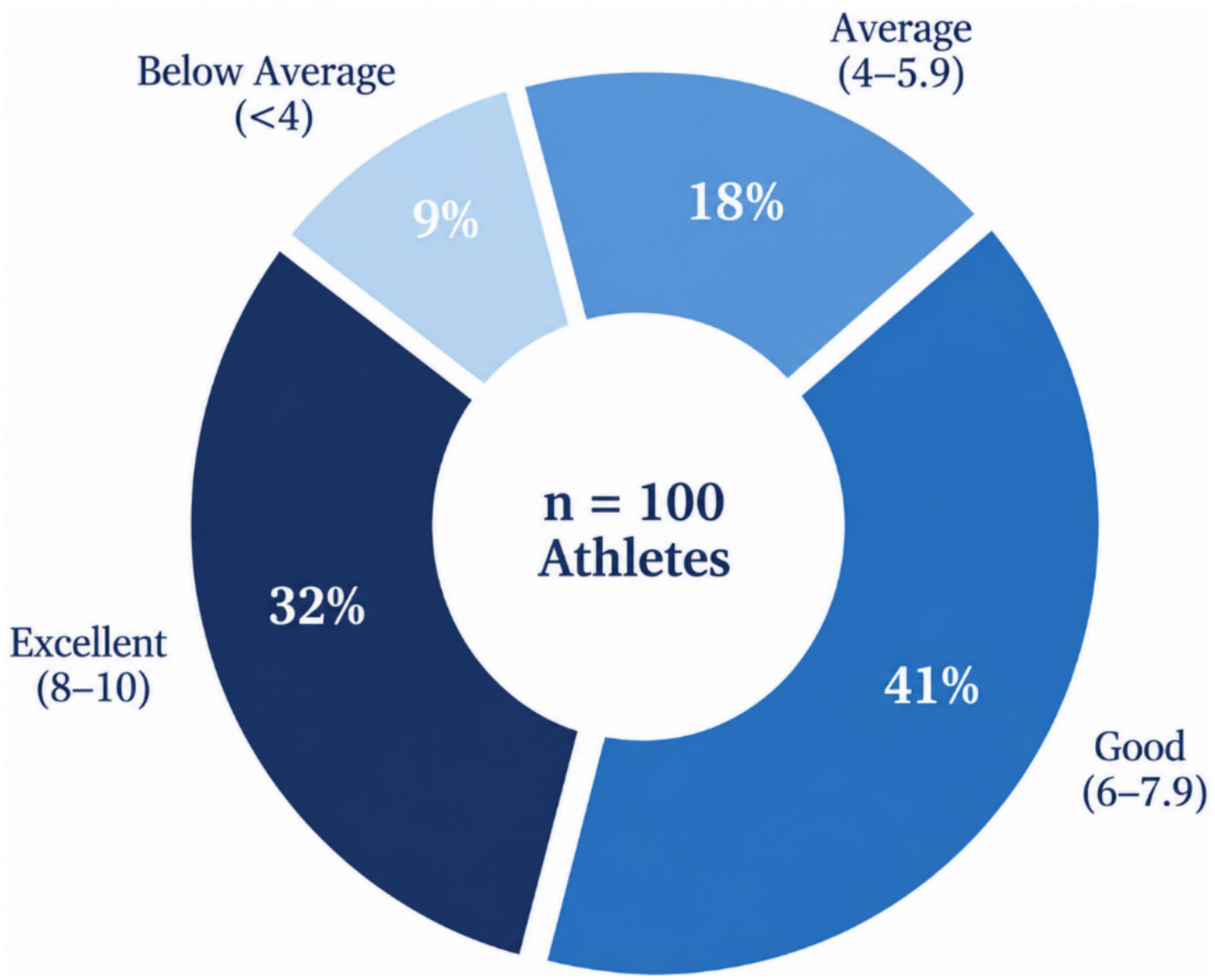
Distribution of athlete self-perceived performance scores (*n* = 100).

The distribution of performance categories is shown in Figure 3: 41% of athletes fell in the “Good” range and 32% in the “Excellent” range, with 9% below average.

### 3.4 Multiple regression analysis

**Figure 4:**
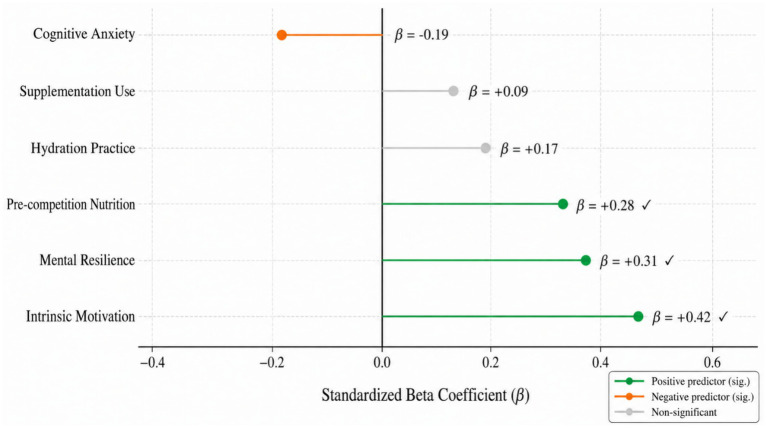
Regression coefficients: predictors of self-perceived performance (*R*^2^ = 0.61).

Six psychological and nutritional constructs were entered as independent variables in a multiple linear regression with self-perceived performance as the dependent variable (25). The model was statistically significant (*F*(6.93) = 24.3, *p* < 0.001) and explained 61% of the variance in performance (*R*^2^ = 0.61, adjusted *R*^2^ = 0.58). Intrinsic motivation was the strongest predictor (*β* = 0.42, *p* < 0.001), followed by mental resilience (*β* = 0.31, *p* < 0.001) and pre-competition nutrition (*β* = 0.28, *p* = 0.001) (26). Cognitive anxiety was a significant negative predictor (*β* = −0.19, *p* = 0.008). Hydration practice approached significance (*β* = 0.17, *p* = 0.062), and supplement use was not significant (*β* = 0.09, *p* = 0.371). Standardized coefficients are displayed in Figure 4.

### 3.5 Moderation analysis: nutritional adherence and the stress–performance association

**Figure 5:**
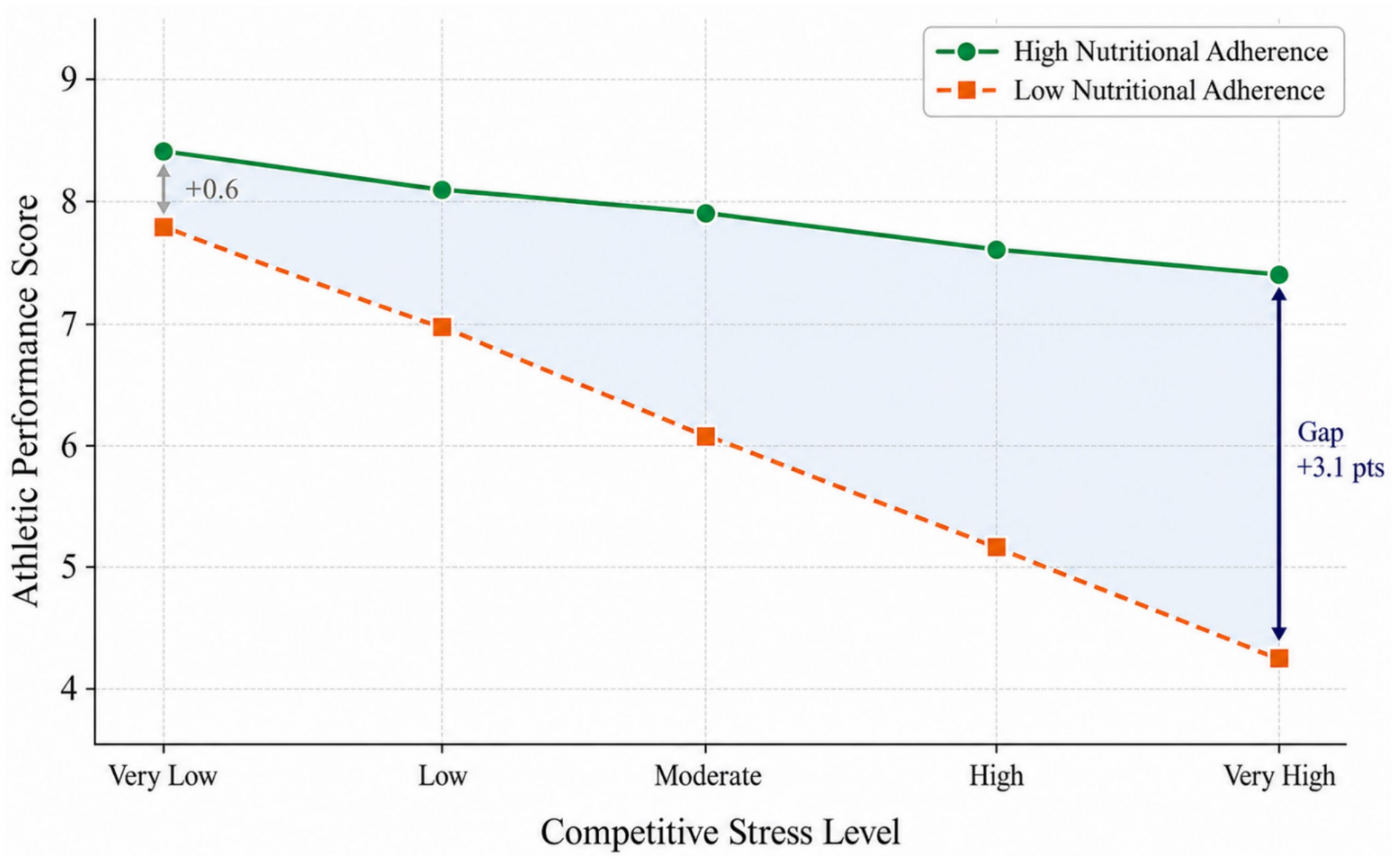
Moderation effect: nutritional adherence moderates the stress–performance association (interaction *β* = −0.23, *p* = 0.014).

A moderation analysis tested whether nutritional adherence moderated the association between competitive stress (predictor) and self-perceived performance (outcome) (13). The interaction term was significant (*β* = −0.23, *p* = 0.014), indicating that the negative association between stress and performance was weaker among athletes reporting higher nutritional adherence. As shown in Figure 5, at low competitive stress the high- and low-adherence groups performed similarly (difference ≈ 0.6 points) (27). As stress increased, the trajectories diverged: athletes with high nutritional adherence showed a modest decline (from 8.4 to 7.4), whereas those with low adherence showed a steeper decline (from 7.8 to 4.3). The largest difference occurred at the highest stress level, where the gap reached 3.1 points (28).

## 4 Discussion

This cross-sectional study examined how psychological factors and nutritional practices relate to self-perceived athletic performance within a single sports organization (29). The findings indicate that motivational and self-regulatory factors, together with self-reported dietary practices, were jointly associated with performance, and that nutritional adherence moderated the association between competitive stress and performance (30).

### 4.1 Psychological factors and self-perceived performance

Intrinsic motivation was the strongest independent correlate of self-perceived performance (*β* = 0.42), supporting the interpretation that motivated athletes are more likely to persist in training, maintain engagement, and invest effort during demanding competitive periods (31). Mental resilience emerged as a further independent correlate (*β* = 0.31), indicating that the ability to recover from setbacks and maintain concentration under pressure was closely related to perceived performance (32). The negative association between cognitive anxiety and performance (*β* = −0.19) suggests that excessive worry before competition may impair focus, decision-making, and execution (33). Taken together, these associations are consistent with and extend to a single organizational setting prior evidence that motivational and self-regulatory factors relate to competitive performance.

### 4.2 Nutritional practices and self-perceived performance

Self-reported pre-competition nutrition was a significant independent correlate of performance (*β* = 0.28, *p* = 0.001), consistent with sports-nutrition research indicating that well-structured pre-competition strategies such as carbohydrate availability (34), protein timing, and micronutrient balance are associated with endurance, cognitive function, and recovery (35). The non-significant association for supplement use (*β* = 0.09) may reflect heterogeneous and unsystematic supplementation within the sample, underscoring the value of protocol-based, professionally supervised supplementation rather than *ad hoc* use.

The moderation finding is of particular interest: higher nutritional adherence was associated with a smaller decline in performance at higher stress levels (36). This pattern is consistent with the interpretation that adequate nutrition functions as a behavioral resource associated with greater stability of cognitive and motor performance under competitive stress, complementing psychological skills rather than substituting for them (37). Because the data are cross-sectional, however, this interpretation should be regarded as an association rather than a demonstrated buffering mechanism, and warrants confirmation in longitudinal or experimental designs (38).

### 4.3 Integrated implications for the organization

Collectively, the findings support a multidisciplinary, integrated approach to performance support. Because psychological and nutritional factors were each associated with performance and because nutritional adherence was associated with attenuated stress-related performance declines interventions targeting mental skills and dietary practices may be most informative when delivered together rather than in isolation. Combining motivational and mental-skills strategies with structured nutritional education and individualized dietary planning is therefore a plausible direction for organization-level support, to be tested in future controlled studies (39).

### 4.4 Limitations and directions for future research

Future research should use longitudinal or intervention-based designs to clarify the direction of associations among psychological factors, nutritional practices, and athletic performance. Future studies should also combine self-reported performance with objective indicators, such as competition results, coach ratings, training-load data, and recovery markers. More detailed nutritional assessment, including dietary intake, hydration status, supplement type and dosage, and sports-nutrition knowledge, would further strengthen interpretation. To improve replication, future studies should clearly report the source, access route, scoring method, and adaptation procedures for standardized scales, and should provide sufficient details for any study-developed instruments, including domains, item structure, response format, and scoring approach.

## 5 Conclusion

Within this defined sports organization, psychological and nutritional factors were jointly associated with athletes’ self-perceived performance, with motivation and mental resilience the most prominent psychological correlates and pre-competition nutritional practices a meaningful behavioral correlate. Beyond confirming that these domains relate to performance, the findings indicate that they are most informative when considered together rather than in isolation, and that nutritional adherence may be associated with a degree of resilience to competitive stress. For practitioners, these results support integrated, organization-level support that combines motivational and mental-skills strategies with structured nutritional guidance, rather than treating psychological and dietary support as separate provisions. Because the evidence is cross-sectional and based on self-report, these implications should be regarded as directions for practice and further investigation rather than established causal effects; confirming them through longitudinal and objectively measured studies represents an important next step.

## Data Availability

The raw data supporting the conclusions of this article will be made available by the authors, without undue reservation.
